# Report of Proceedings of the Pennsylvania Society of Dental Surgons

**Published:** 1850-01

**Authors:** 


From the Dental News Letter.

### REPORT OF PROCEEDINGS OF THE PENNSYLVANIA SOCIETY OF DENTAL SURGONS.

A stated meeting of the society was held December fourth, 1849, at the usual place. Mr. C. C. Williams, President in the chair. A. R. Johnson, Secretary. After the usual preliminary business, Mr. J. McQuillan was elected to membership.

An essay from Dr. Jas. Fleming, was read by the Secretary; after which, on motion of Mr. S. S. White, a vote of thanks was returned to Dr. Fleming; also, that a copy be furnished the proprietors of the "News Letter" for publication.

A communication from Mr. F. Reinstein, was now read; the subject—caries. In some prefatory remarks, he says, "On establishing the society, it was forcibly impressed upon our minds, that social intercourse, and the imparting to each other the knowledge obtained in the daily practice of our profession, would add greatly to the advancement to each individual member in the dental art.

"It is highly necessary that we awake to the importance of improvement, and, that in future, every one of us, to push on the good work, ought to bring, in turn, something before the society, no matter how small, in which our views or practice is given.

"Our art opens for us a wide field for observation, on which to write or speak, and those who have, by experience, a better view of the case related to them, should meet the other with kindness, to show him his error, if any. The most timid, would in this way gain confidence, and be encouraged to collect and arrange his ideas on the subject which he may wish to bring before the society.

"By these means, the great object of our society will be accomplished, and ourselves bound more closely by the ties of fellowship."

Oral communications being now in order, Dr. J. D. White made a few remarks on amalgams, condemning their use, on account of the mercury they contain, and their contraction in hardening, etc.; giving it as his opinion, that it was not necessary that there should be oxydation of the metals to be detrimental to the teeth.

On motion, the Librarian was authorized to subscribe for the "Dental Register of the West."

Messrs. Jas. Parry and A. R. Johnson, were appointed to deliver essays at next meeting. After the transaction of some important business—adjourned.

